# Distinct Activation Mechanisms of NF-κB Regulator Inhibitor of NF-κB Kinase (IKK) by Isoforms of the Cell Death Regulator Cellular FLICE-like Inhibitory Protein (cFLIP)*

**DOI:** 10.1074/jbc.M116.718122

**Published:** 2016-02-10

**Authors:** Mehdi Baratchian, Christopher A. Davis, Akira Shimizu, David Escors, Claire Bagnéris, Tracey Barrett, Mary K. Collins

**Affiliations:** From the ‡ Medical Research Council Centre for Medical Molecular Virology, Division of Infection and Immunity, University College London, London WC1E 6BT, United Kingdom,; § Division of Advanced Therapies, National Institute of Biological Standards and Control, Blanche Lane, South Mimms, Potters Bar, Herts EN6 3QG, United Kingdom, and; ¶ Institute of Structural and Molecular Biology, School of Biological Sciences, Birkbeck College, Malet Street, London WC1E 7HX, United Kingdom

**Keywords:** cell signaling, death domain, NF-κB, protein kinase, ubiquitylation (ubiquitination)

## Abstract

The viral FLICE-like inhibitory protein (FLIP) protein from Kaposi sarcoma-associated herpesvirus activates the NF-κB pathway by forming a stable complex with a central region (amino acids 150–272) of the inhibitor of NF-κB kinase (IKK) γ subunits, thereby activating IKK. Cellular FLIP (cFLIP) forms are also known to activate the NF-κB pathway via IKK activation. Here we demonstrate that cFLIP_L_, cFLIP_S_, and their proteolytic product p22-FLIP all require the C-terminal region of NEMO/IKKγ (amino acids 272–419) and its ubiquitin binding function for activation of the IKK kinase (or kinase complex), but none form a stable complex with IKKγ. Our results further reveal that cFLIP_L_ requires the linear ubiquitin chain assembly complex and the kinase TAK1 for activation of the IKK kinase. Similarly, cFLIP_S_ and p22-FLIP also require TAK1 but do not require LUBAC. In contrast, these isoforms are both components of complexes that incorporate Fas-associated death domain and RIP1, which appear essential for kinase activation. This conservation of IKK activation among the cFLIP family using different mechanisms suggests that the mechanism plays a critical role in their function.

## Introduction

The first members of the FLICE^2^-like inhibitory protein (FLIP) family were identified as viral genome products (vFLIPs) following a bioinformatics search conducted to recognize death effector domain-containing proteins (1). A consensus sequence from the viral FLIPs was then used to screen human expressed sequence tags, and several human homologs were identified, now known collectively as cellular FLIPs (cFLIPs) (for a review, see Ref. 2). Cellular FLIPs are encoded by a single gene; in humans there are three main splice variants, long isoform (cFLIP_L_; 55 kDa), short isoform (cFLIP_S_; 26 kDa), and cFLIP Raji (cFLIP_R_; 24 kDa) first detected in the human Burkitt lymphoma B cell line Raji (3). Mice do not express cFLIP_S_ and contain only two isoforms cFLIP_L_ and cFLIP_R_ (4).

All cFLIP isoforms share an identical N terminus of 202 amino acids, which includes two death effector domains, but have different C termini. The structure of cFLIP_S_ is similar to the vFLIPs, but it contains a 19-amino acid C terminus that is responsible for its ubiquitination and proteasomal degradation (5). In cFLIP_L_, the tandem death effector domains are followed by a caspase-like domain. In addition, two N-terminal cleavage products have been detected, p43-FLIP and p22-FLIP, both thought to be involved in stimulating NF-κB signaling (6, 7). The best described function of cellular FLIPs is in regulation of death receptor (DR) signaling. Procaspase-8 activation, which is pivotal for DR-induced apoptosis, occurs through an “induced proximity” mechanism where DR-mediated homodimerization of procaspase-8 leads to its self-cleavage and activation. Like procaspase-8, cFLIPs are recruited to the DR complex by FADD and can thus directly inhibit procaspase-8 activation (8). However, cFLIP_L_ in particular also contributes to DR-induced NF-κB signaling (9–11), particularly when expressed at a moderate level (12).

The canonical NF-κB pathway is activated by inhibitor of NF-κB kinase (IKK), which phosphorylates IκB, leading to its degradation. This releases NF-κB transcription factors, which then activate gene expression. vFLIP is a latently expressed gene from Kaposi sarcoma-associated herpesvirus (KSHV), which activates NF-κB signaling (13). NF-κB activation by vFLIP leads to the characteristic phenotype and cytokine secretion of Kaposi sarcoma cells (14) and survival of KSHV-transformed B cells (15–17). vFLIP activates IKK (18) by forming a stable complex with the regulatory subunit IKKγ (19). We have solved the crystal structure of two vFLIP molecules bound to a parallel α-helical coiled coil of two IKKγ molecules and proposed a mechanism whereby this binding leads to activation of the IKKα/β kinase subunits (20).

In this study, we wished to investigate how cFLIPs activate IKK and in particular whether they form a stable complex with IKK in a similar manner to vFLIP. We therefore engineered cells to express the various cFLIP isoforms and measured their ability to activate IKK in the absence of any additional signal. In contrast to previous reports, we did not find any direct interaction with IKKγ. The ubiquitin binding function of IKKγ was required by all the cFLIP variants. In the case of cFLIP_L_, the linear ubiquitination complex LUBAC was necessary, presumably to generate a ubiquitinated substrate to interact with IKKγ. In contrast, cFLIP_S_ and p22-FLIP formed a complex including FADD and RIP1 that led to RIP1 ubiquitination and activation of IKK. These data are relevant to a variety of human cancers as we have demonstrated that human tumor cells express a number of cFLIP variants at levels similar to those in our engineered cells.

## Experimental Procedures

### 

#### 

##### Reagents and Antibodies

Recombinant human TNF-α (PeproTech, Rocky Hill, NJ), ultrapure LPS and hygromycin B (InvivoGen, San Diego, CA), 10 mm ATP solution (Life Technologies), and [γ-^32^P]ATP (SRP-501, Hartman Analytic, Germany) were from the indicated sources. Antibodies against caspase-8 (C-20), HA tag (Y-11), IKKα/β (H-470), NEMO/IKKγ (FL-419), and TAK1 (M-579) were from Santa Cruz Biotechnology. Mouse monoclonal antibodies for FADD (1/FADD) and RIP1 (38/RIP) were obtained from BD Biosciences. Antibodies specific for Atg3, calnexin (C5C9), GAPDH (14C10), MEKK3 (D36G5), pIκBα (14D4), and pIKKα/β (C84E11) were from Cell Signaling Technology. Anti-FLAG (M2), anti-HOIP (SAB2701544), and anti-CYLD (SAB4200061) were from Sigma-Aldrich. Anti-cFLIP (NF6) was from Enzo Life Sciences, and anti-cFLIP (G11) was from Santa Cruz Biotechnology. Antibodies recognizing HOIL-1 and SHARPIN were generously provided by Prof. Henning Walczak (University College London Cancer Institute), and human tumor cells lines were from Dr. Pablo Rodriguez-Viciana (University College London Cancer Institute). HRP-coupled sheep anti-mouse IgG (GE Healthcare), rabbit anti-goat IgG (Dako), and Fc fragment-specific goat anti-rabbit and light chain-specific mouse anti-rabbit IgG (both from Jackson ImmunoResearch Laboratories) were obtained from the indicated sources.

##### Cell Culture and Transfections

HEK293 and 293T cells were grown in DMEM with 10% FCS, 2 mm l-glutamine, 100 μg/ml streptomycin, and 100 units/ml penicillin. 70Z/3 and 1.3E2 cells (Ref. 21; kind gifts from Prof. Alain Israel) were grown in RMPI 1640 medium with FCS/glutamine/penicillin/streptomycin and 50 μm 2-mercaptoethanol.

##### Lentivectors and Cell Transductions

**FIGURE 1.**
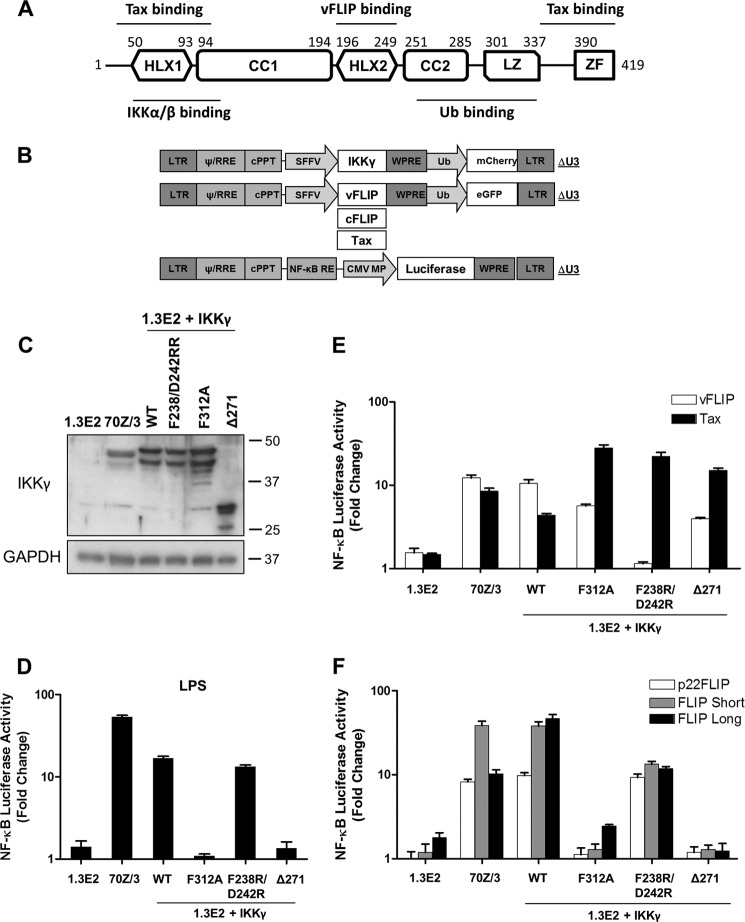
**Mutation of IKKγ reveals different NF-κB activation mechanisms by KSHV vFLIP and cellular FLIPs.** *A*, schematic representation of IKKγ and the regions involved in interaction with IKKα/β, KSHV vFLIP, Tax, and ubiquitin (*Ub*). *CC*, coiled coil; *HLX*, helical domain; *LZ*, leucine zipper; *ZF*, zinc finger. *B*, map of the lentivectors. *CMV MP*, minimal promoter of cytomegalovirus; *cPPT*, central polypurine tract; *LTR*, long terminal repeat; *NF-*κ*B RE*, NF-κB response element; *RRE*, rev response element; *SFFV*, spleen focus-forming virus; *WPRE*, woodchuck post-transcription regulatory element. *C*, immunoblot showing the expression of IKKγ in 1.3E2 cells reconstituted with wild-type or mutant IKKγ. The blot was reprobed for GAPDH. *D*, to generate stable NF-κB reporter cell lines, cells were transduced with lentivectors encoding an NF-κB-responsive luciferase gene. The luciferase reporter assays were performed 6 h after stimulation with LPS (10 μg/ml) or 48 h following transduction with lentivectors encoding vFLIP, Tax (*E*), and cFLIP variants (*F*). Data are representative of three independent experiments performed in triplicate. *Error bars* indicate S.D. of the mean values.

cDNA of the non-cleavable cFLIP_L_ was a kind gift from Prof. Inna Lavrik. cDNAs of wild-type or mutant FLIP variants, Tax, and N-terminally HA-tagged FADD were cloned into pDual (see Fig. 1*B*). Vesicular stomatitis virus G glycoprotein pseudotyped lentiviral particles were produced by co-transfection of HEK293T cells with pDual, pCMV8.91 (HIV gag-pol), and pMDG (vesicular stomatitis virus G glycoprotein). Supernatants were passed through 0.45-μm filters and concentrated 100-fold by ultracentrifugation at 100,000 × *g* at 4 °C for 2 h in a Sorvall centrifuge (Beckman Coulter). Viral pellets were resuspended in cold whole medium, incubated on ice for 1–2 h, aliquoted, and stored at −80 °C. Titers were measured on HEK293T cells by fluorescence-activated cell sorting (FACS) analysis and TaqMan quantitative PCR (Applied Biosystems, Warrington, UK). For experiments, cells were transduced with test cFLIP, vFLIP, and Tax lentivectors at identical multiplicities of infection within experiments and in the range of 1–20 or 50–500 for 293/293T or 70Z/3, respectively.

##### Generation of Stable Knock-down Cell Lines

shRNA-expressing lentivectors targeting HOIP, TAK1, Atg3, caspase-8, RIP1, and FADD were produced using pGIPZ lentivector plasmids (University College London Open Biosystems shRNA library, London, UK) that contain a puromycin resistance gene and a GFP marker. For silencing of HOIL-1 and SHARPIN, we designed customized shRNA-encoding DNA duplexes using the Clontech online RNAi designer tool and cloned them into the pSIREN Hygro lentivector backbone (modified from pSIREN RetroQ; Clontech). The transduced cells were selected with hygromycin B (200 μg/ml). The sequences targeted for each gene have been listed in Table 1.

**TABLE 1:** **shRNA-targeted sequences of the silenced proteins in this study**

Gene symbol	NCBI Ref Seq no.	Targeted sequence
*HOIL-1*	NM_006462.4	CCACAACACTCATCTGTCAAA
*HOIP*	NM_017999.4	AGCTGCTGTGCTATGTTCC
*SHARPIN*	NM_030974.3	CTGTCCTTCCTGCACCTTCAT
*TAK1*	NM_003188.3	AGAGTGAATCTGGACGTTT
*MEKK3*	NM_203351.1	CCTGGATATGAGACCATGA
*Atg3*	NM_022488.4	1, CATTGACCATATTCATCAA
		2, GGACTTATATGTTTATGCA
Caspase-8	NM_001228.4	1, TGCACAGTAGAGCAAATCT
		2, GGGTGGTTATTGAAAGTAA
*FADD*	NM_003824.3	1, CAGGACGAATTGAGATAAT
		2, AAGATCTTGTCTCCACTAA
*RIP1*	NM_003804.3	1, AGAGTAAACTCCAAGACGA
		2, ACCACTAGTCTGACGGATA

##### Immunoblotting, Precipitation, and Kinase Assays

Cell extracts were prepared with lysis buffer (1% Triton X-100 in 20 mm Tris-HCl, pH7.5, 150 mm NaCl, and 5% glycerol supplemented with phosphatase and protease inhibitors). Lysates (20–40 μg/lane) were separated on 8–12% SDS-polyacrylamide gels and transferred to PVDF membranes (GE Healthcare), which were then blocked with 5% skimmed milk in TBS-T (0.1% Tween 20 in 50 mm Tris-HCl, pH 7.6, and 150 mm NaCl) for 1 h. Membranes were incubated overnight at 4 °C with suitable dilutions of primary antibodies in TBS-T buffer containing 5% BSA and 0.1% sodium azide. Bound antibodies were detected with appropriate peroxidase-conjugated secondary antibodies and visualized by the ECL system (LumiGLO, Cell Signaling Technology). For immunoprecipitation, lysates were first precleared by incubating with Protein A- or G-conjugated beads (Sigma) for 30 min at 4 °C. The precleared extracts (300–500 μg) were then incubated with 1 μg of primary antibody or its isotype-matched IgG control and 20 μl of Sepharose beads for 2–4 h at 4 °C. The beads were washed twice with high salt wash buffer (25 mm Tris-HCl, pH7.6, 500 mm NaCl, 1 mm DTT, and 5% glycerol) and two additional times with the lysis buffer and then were resuspended in 20 μl of 2× Laemmli buffer. IKK kinase assays were performed on immune complexes as described previously (19). The reaction mixtures were separated by 10% SDS-PAGE, transferred to a PVDF membrane, and exposed to a phosphor screen for 15–30 min. The radiolabeled phosphoproteins were then visualized using a Typhoon phosphorimaging system (GE Healthcare) and analyzed by ImageQuant TL Plus 7.0 software (GE Healthcare).

##### In Vitro IκBα Phosphorylation Assay

Resting HEK293T cells were lysed in HTX lysis buffer (10 mm Hepes, pH 7.4, 10 mm MgCl_2_, 1 mm MnCl_2_, 0.1 mm EGTA, and 0.5% Triton X-100) supplemented with complete protease inhibitor mixture (Roche Applied Science), and the S100 fractions were prepared by sequential centrifugation at 1,000 and 100,000 × *g* for 5 and 45 min, respectively. The cleared lysates were then incubated with recombinant FLIP and an ATP-generating system (10× stock: 10 mm ATP, 20 mm Hepes, pH 7.2, 10 mm MgCl_2_, 350 μm creatinine phosphate, and 500 μg/ml creatinine kinase) for 10 min at 37 °C. The reactions were terminated by adding 5× Laemmli buffer and boiling at 95 °C. The recombinant FLIP-induced IKK activation rate was detected by immunoblotting for pIκBα and pIKKα/β.

##### Purification and Expression of Recombinant vFLIP, p22-FLIP, and GB1-p22-FLIP

Recombinant vFLIP was overexpressed and purified as described in Bagnéris *et al.* (20). For the production of recombinant p22-FLIP (and GB1-p22-FLIP), cFLIP_S_ was first PCR-amplified from a human thymus cDNA library (Clontech) using the primers 5′-TTGCTAGCATGTCTGCTGAAGTC and -TTCTCGAGTCACATGGAACAATTTC containing NheI and XhoI restriction sites, respectively (underlined). Following digestion, the product was then ligated into the pETM442 vector (described in Bagnéris *et al.* (20)) to provide an N-terminal His_6_-NusA tobacco etch virus protease-cleavable solubility tag to aid expression and purification. The resulting pETM442-cFLIP_S_ vector was then used as the template for production of pETM442-p22-FLIP, which was generated by the introduction of a stop codon at Asp-196 using the primers TCCAAAAGAGTCTCAAGTAGCCTTCAAATAACTTCAGGAT and ATCCTGAAGTTATTTGAAGGCTACTTGAGACTCTTTTGGG. GB1-p22-FLIP was produced by subcloning p22-FLIP into the EcoRI and XhoI restriction sites of a modified pET15b vector (a kind gift from Prof. Paul Driscoll, National Institute for Medical Research) to produce pET15-GB1-p22-FLIP (containing a His_6_-GB1 solubility tag). The original pET15b vector was modified by inclusion of the coding sequence for the 56-amino acid immunoglobulin domain of *Escherichia coli* protein G (GB1) 5′ to the multiple cloning site, the addition of an EcoRI restriction site directly 3′ to GB1, and removal of the multiple cloning site EcoRI site. This enabled subcloning of p22-FLIP 3′ to GB1 into the new EcoRI and existing XhoI sites. pETM442-p22-FLIP and pET15-GB1-p22-FLIP were then transformed into BL21(DE3) Star^TM^ cells (Invitrogen). The cultures were then inoculated into LB medium containing ampicillin (100 μg/ml) at a ratio of 1:100 and grown to an *A*_600_ of between 0.6 and 0.8. Cultures were subsequently induced with 1 mm isopropyl 1-thio-β-d-galactopyranoside, and the temperature was reduced to 16 °C overnight. Cells were harvested by centrifugation, and the pellets were either frozen at −80 °C or resuspended in a buffer comprising 25 mm Tris-HCl, pH 8.5, and 200 mm NaCl (buffer A) supplemented with an EDTA-free protease inhibitor complex tablet (Roche Applied Science) and DNase I (10 μg/ml). The purification protocols used for p22-FLIP and GB1-p22-FLIP were nearly identical. Resuspended pellets from 8-liter cultures of both proteins were sonicated on ice, and the lysates were clarified by centrifugation (46,000 × g for 1 h). Supernatants were subsequently filtered through a 0.45-μm filter prior to loading on HisTrap FF columns (GE Healthcare) pre-equilibrated with buffer A. These were then washed with buffer B (buffer A and 20 mm imidazole) and eluted with buffer C (buffer A and 500 mm imidazole). For p22-FLIP, the His_6_-NusA tag originating from the pETM442-p22-FLIP construct was removed by incubating His_6_-NusA-p22-FLIP with tobacco etch virus protease overnight while dialyzing in buffer A (to remove the imidazole), and the solution was reapplied to a 5-ml HisTrap FF column pre-equilibrated in buffer A. p22-FLIP was eluted from the column using buffer A and 40 mm imidazole. Fractions having greater than 95% purity as judged by SDS-PAGE were subsequently pooled and frozen at −80 °C. Following elution from the 5-ml HisTrap column, GB1-p22-FLIP appeared to be over 95% pure. To improve stability, however, GB1-p22-FLIP was subsequently dialyzed into a buffer containing 5 mm l-arginine, 300 mm NaCl, 5 mm tris(2-carboxyethyl)phosphine, and 25 mm imidazole prior to concentration using a 6-ml, 10-kDa-molecular mass cutoff Vivaspin concentrator (Millipore) and storage at −80 °C.

##### p22-FLIP Pulldowns with IKKγ

Pulldowns were performed using His_6_-IKKγ(40–419) immobilized to a 1-ml nickel-nitrilotriacetic acid column (GE Healthcare) and purified p22-FLIP as described for vFLIP in Bagnéris *et al.* (20).

##### NF-κB Luciferase Reporter Assays

For some reporter assays, cells were transfected with 300 ng of NF-κB firefly luciferase plasmid (pGL immunoglobin κ light chain (IgK) or pGL H2-Dk), 100 ng of pRL.TK as an internal control, and 500 ng of pcDNA3 vectors encoding NF-κB activators. 24 h later, luciferase activity was measured using a Dual Glo Luciferase kit (Promega). Firefly luciferase activity levels were normalized to that of *Renilla*. Alternatively, HEK293/293T and 70Z/3 cells were transduced with NF-κB firefly luciferase lentivectors at multiplicities of infection of 10 and 500, respectively. NF-κB-induced luminescence was detected using a Varioskan Flash multimode reader (Thermo Scientific).

##### Statistical Analysis

Statistical differences between two groups were analyzed by the two-tailed unpaired Student's *t* test using the GraphPad Prism v4.03 software package (GraphPad Software, La Jolla, CA). The calculated *p* values are given in the figures. All experiments were performed in triplicates on at least three independent occasions. The quantitative data are presented as mean ± S.D.

## Results

### 

#### 

##### cFLIP Isoforms Require the Ubiquitin Binding Function of IKKγ

To determine which functions of IKKγ were necessary for IKK activation by cFLIP isoforms, we used the IKKγ-null cell line 1.3E2 (21) harboring an NF-κB-responsive luciferase gene. These cells were reconstituted with a truncated IKKγ lacking the ubiquitin binding domain (Δ271), a point mutant that does not bind linear ubiquitin (F312A (22)), or a mutant defective in vFLIP binding (F238R/D242R (23)) (Fig. 1*A*). To do this, we used lentivectors expressing wild-type or mutant IKKγ together with an mCherry fluorescent protein (Fig. 1*B*). Cells were transduced so that about 50% were mCherry-positive. We then isolated cell clones, expanded those that were mCherry-positive, and performed immunoblotting to establish that the transduced cells expressed IKKγ (Fig. 1*C*). Cells were then infected with a second lentivector expressing cFLIP variants, vFLIP, or Tax from human T lymphotropic virus, type 1, another known IKK activator that binds directly to IKKγ (24), together with enhanced GFP. After 48 h, enhanced GFP was monitored, and cells with transduction rates over 80% were used to measure luciferase activity.

Fig. 1*E* shows that both vFLIP and Tax could activate IKK in the absence of its ubiquitin binding function, unlike the control Toll-like receptor agonist lipopolysaccharide (Fig. 1*D*). Tax has been reported to interact with two regions of IKKγ at both the N and C termini (Ref. 23 and shown in Fig. 1*A*). However, our data demonstrate that only the N-terminal interaction is necessary for IKK activation. It was also reported previously that vFLIP can activate IKK reconstituted with IKKγ truncated at amino acid 251 (25). In contrast, Fig. 1*F* shows that cFLIP_L_, cFLIP_S_, and their proteolytic product p22-FLIP all require the ubiquitin binding domain of IKKγ and its ubiquitin binding function to activate IKK but do not require the residues that interact with vFLIP.

**FIGURE 2.**
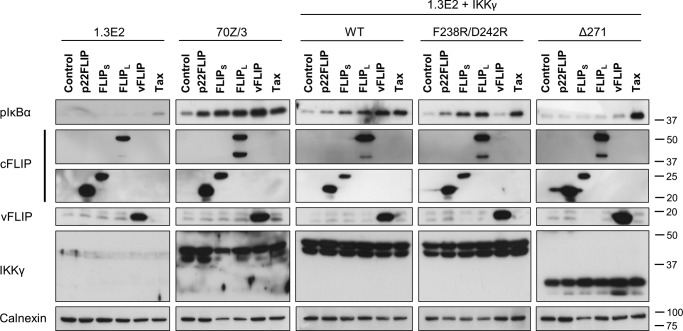
**Cellular FLIPs activate vFLIP binding-deficient IKKγ.** 1.3E2, 70Z/3, and 1.3E2 cells stably expressing IKKγ variants were transduced with lentivectors encoding cFLIP variants, vFLIP, and Tax. 48 h post-transduction, cells were treated with the proteasome inhibitor MG-132 (5 μm) for 30 min and then lysed in cell lysis buffer. The cytoplasmic extracts were analyzed for NF-κB activation by immunoblotting with an antibody that recognized phospho-IκBα.

To confirm these data, we examined the phosphorylation of IκBα in the same cell lines. Fig. 2 shows that vFLIP and Tax could stimulate phosphorylation of IκBα when IKKγ lacked the ubiquitin binding domain in contrast to all the cFLIP isoforms. We also observed that Tax could stimulate phosphorylation of IκBα in cells lacking IKKγ (Fig. 2), and therefore Tax gave a higher stimulation of phosphorylation of IκBα when IKKγ lacked the ubiquitin binding domain than did vFLIP. This was observed previously, although the mechanism remains unexplained (21).

##### cFLIP Isoforms Generate an Active IKK without Stable Interaction with IKKγ

**FIGURE 3.**
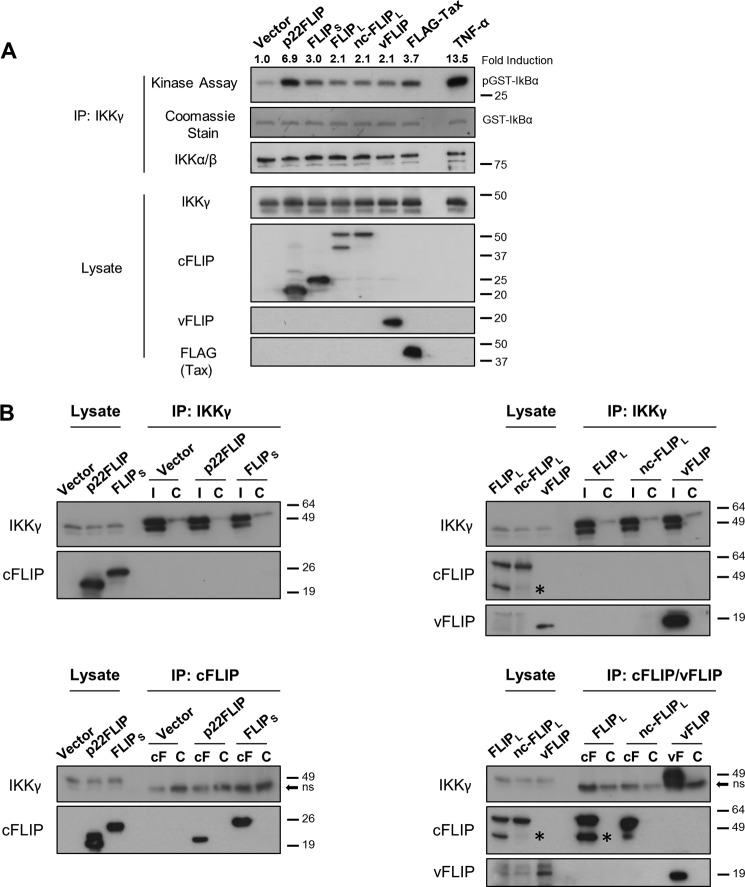
**Cellular FLIPs constitutively activate IKK complex without stable association with IKKγ.** *A*, *in vitro* kinase assays to determine the activation of IKK. HEK293T cells were transfected with pcDNA3 vectors encoding p22FLIP, FLIP_S_, FLIP_L_, non-cleavable FLIP_L_, vFLIP, or Tax. 48 h later, whole cell lysates were extracted and subjected to immunoprecipitation (*IP*) with anti-IKKγ or immunoblotting (*IB*) with the indicated antibodies. An *in vitro* kinase assay was then performed on immunoisolated IKK complexes using GST-IκBα(1–54) and [γ-^32^P]ATP as substrates. An extract of cells treated with TNF-α (10 ng/ml) for 5 min was used as a positive control. Relative band intensity of phosphorylated GST-IκBα (*pGST-I*κ*B*α) was quantified by ImageQuant TL Plus 7.0 and normalized to the corresponding immunoprecipitated IKKα/β levels. *B*, cFLIP variants are not found in complex with IKKγ. HEK293T cells were transfected with pCDNA3 constructs using FuGENE HD transfection reagent. Cells were lysed 48 h later, and extracts were immunoprecipitated with 2 μg of antibodies against IKKγ, cFLIP, and vFLIP or their isotype-matched controls. The immunoprecipitates were subject to 10% SDS-PAGE and analyzed by immunoblotting. Data shown are representative of at least four independent repeats. * indicates the position of p43-FLIP bands. *cF*, cFLIP; *C*, isotype control; *I*, IKKγ; *ns*, nonspecific; *vF*, vFLIP.

**FIGURE 4.**
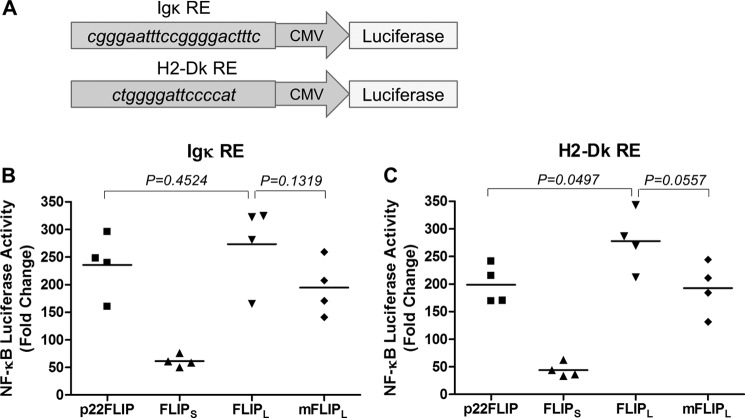
**Non-cleavable mutant of cFLIP_L_ activates NF-κB pathway in levels comparable with that of wild-type cFLIP_L_.** *A*, two different NF-κB luciferase plasmids were constructed to compare the activates of the cFLIP variants: one with four repeats of NF-κB response element (*RE*) of IgK gene and another with four repeats of an NF-κB binding site of the mouse MHC class I H2-Dk gene. *B*, NF-κB luciferase assay comparing the NF-κB activation levels induced by WT or mutant FLIP_L_ (*mFLIP_L_*). The luciferase reporter assays were performed as described in Fig. 5*C*. Results shown are representative of four independent experiments, and individual values are shown with a *horizontal bar* representing the mean.

To examine the mechanism of activation of IKK by cFLIP isoforms, we transfected HEK293T cells with expression plasmids for cFLIP_S_, cFLIP_L_, a non-cleavable version of cFLIP_L_ (cFLIP_L_ D196E/D376N), and p22-FLIP as well as vFLIP as a positive control for IKK direct interaction. We then immunoprecipitated IKK using an anti-IKKγ antibody and measured its ability to phosphorylate recombinant IκBα. Fig. 3*A* shows that in each case the cFLIP isoforms generated an activated IKK. The level of activation was comparable or greater than that generated by vFLIP although less than the transient activation observed following TNF-α treatment (Fig. 3*A*). In contrast to a previous report, we observed a similar level of activation with non-cleavable cFLIP_L_ as with wild type (6). As another report had used an NF-κB luciferase reporter with an H2-Dk response element to measure the activity of cFLIPs (7), we also tested this NF-κB element. Fig. 4 shows that the non-cleavable cFLIP_L_ was no different in activity from wild type with either reporter. Note that cFLIP_S_ generally gave a relatively lower level of stimulation in HEK293 and 293T cells, which we cannot explain. We then examined whether the cFLIP isoforms were found associated with the activated kinase and found no evidence for the presence of cFLIP isoforms in the IKK immunoprecipitates, although vFLIP was clearly present (Fig. 3*B*). We also demonstrated that recombinant p22-FLIP does not form a stable complex with recombinant IKKγ (data not shown) unlike vFLIP (20). These data are in contrast to the previous reports that showed p22 and p43 fragments stably associated with IKK (7, 12). Clearly, differences in the experimental conditions can explain these results; however, our data demonstrate that the cFLIP isoforms can generate an active IKK without remaining physically associated with the IKK complex.

##### All cFLIP Forms Require TAK1, and cFLIP_L_ Requires LUBAC to Activate IKK

**FIGURE 5.**
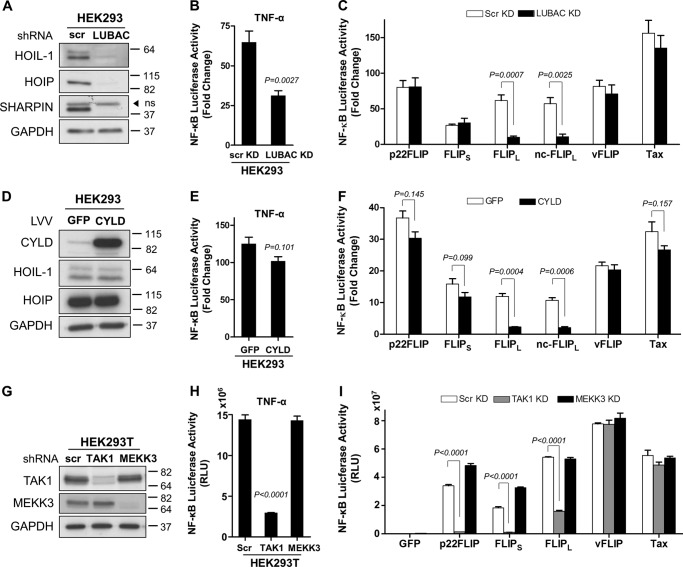
**Only cFLIP_L_ is dependent on LUBAC to induce NF-κB, whereas all cFLIP isoforms require TAK1.** *A*, HEK293 cells were transduced with lentivectors encoding either scrambled control shRNA (*scr*) or shRNAs targeting HOIL-1, HOIP, and SHARPIN sequentially. Generation of LUBAC knockdown (*KD*) cells was confirmed by immunoblotting against each component of the complex and the housekeeping protein GAPDH. *ns*, nonspecific. *B*, TNF-α-induced NF-κB activation which was measured 6 h after stimulation at 10 ng/ml concentration is inhibited in LUBAC knockdown cells. *C*, scrambled and LUBAC knockdown HEK293 cells were co-transfected with an NF-κB firefly luciferase reporter construct (300 ng/well) and a *Renilla* luciferase reporter vector (normalization control, 100 ng/well) with empty or transactivator-expressing pcDNA3 vectors (500 ng/well). The luciferase reporter assay was performed 24 h post-transfection. *D*, immunoblot showing the expression of ectopically expressed CYLD in HEK293 cells. Cells transduced with a GFP-expressing lentivector were used as controls. Effects of CYLD overexpression on NF-κB activation levels induced by TNF-α (*E*) or cFLIPs, vFLIP, and Tax (*F*) were measured as described above. *G*, shRNA-mediated stable silencing of TAK1 and MEKK3 in HEK293T cells was validated by immunoblotting. *H*, these cells were further transduced with NF-κB luciferase lentivector to develop NF-κB sensor cell lines. Luciferase reporter assays were then performed 6 h following stimulation with TNF-α (10 ng/ml) or 48 h following transduction with control (enhanced GFP) or test lentivector (*I*). Results shown are mean ± S.D. (*error bars*) of a representative experiment of three, performed in triplicates. The two-tailed Student's *t* test was used to determine the statistical differences between two groups. *RLU*, relative luciferase units; *LLV*, luciferase lentivector.

Given that the cFLIP isoforms require the ubiquitin binding domain of IKKγ (Figs. 1 and 2), we sought to establish which of the ubiquitination pathways have a key role and whether the cFLIP variants have different requirements for activation. Linear ubiquitin chain binding to IKKγ is crucial for IKK activation by TNF-α (22), and these are generated by the trimeric LUBAC composed of HOIL-1, HOIP, and SHARPIN (26). We therefore generated HEK293 cells that were stably transduced with lentivectors containing short hairpin RNAs targeting HOIL-1, HOIP, and SHARPIN (Fig. 5*A*). As reported previously, LUBAC was required for full IKK activation by TNF-α (26) and was dispensable for activation by vFLIP (27) and Tax (Fig. 5, *B* and *C*). A clear blockade of cFLIP_L_ activation of IKK was observed in the LUBAC knockdown cells (Fig. 5*C*). However, cFLIP_S_ and p22-FLIP were unaffected by LUBAC knockdown (Fig. 5*C*). We also overexpressed in HEK293 cells the deubiquitinase CYLD, which can remove ubiquitin chains with linear as well as KLys-63 linkages (28) (Fig. 5*D*) and which inhibited full IKK activation by TNF-α (Fig. 5*E*). In this case, cFLIP_L_ was once again inhibited, whereas cFLIP_S_ and p22-FLIP were unaffected (Fig. 5*F*). The kinase TAK1 has been identified as a critical activator of IKK, phosphorylating the activation loops of the kinase subunits in response to a variety of signals (29, 30), and the kinase MEKK3 has also been reported to fulfil this role in some circumstances (31). We therefore also produced cells with stably knocked down TAK1 or MEKK3 (Fig. 5*G*). TAK1 was clearly necessary for activation of IKK by all the cFLIP variants, although both kinases were dispensable for vFLIP and Tax activation. The lack of a requirement for TAK1 in vFLIP activation has been reported (27), although its role in Tax activation of IKK has been controversial (32, 33). The requirement for TAK1 in the cFLIP isoform-mediated pathways suggests that the active kinase identified in Fig. 3 has been phosphorylated and activated by TAK1. The lack of requirement for ubiquitin signaling by cFLIP_S_ and p22-FLIP (Fig. 5, *A* and *D*) required further investigation as the ubiquitin binding domain of IKKγ and its ubiquitin binding function were clearly required (Figs. 1 and 2).

##### cFLIP_S_ and p22-FLIP Activate IKK via a FADD·RIP1 Complex

**FIGURE 6.**
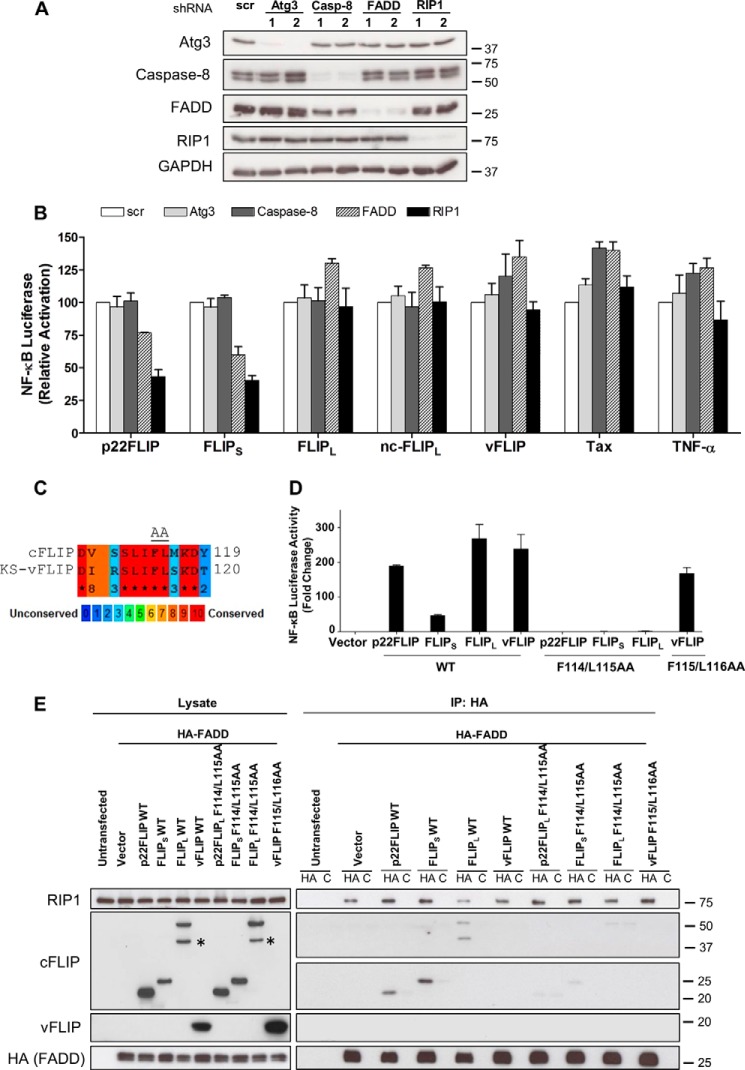
**p22-FLIP and cFLIP_S_ require a FADD·RIP1 complex to induce IKK.** *A*, stable gene knockdown of Atg3, caspase-8 (*Casp-8*), FADD, and RIP1 in HEK293T cells was validated by immunoblotting. From five independent shRNAs used to silence each protein, two that yielded the most efficient knockdowns were chosen for the experiments. Targeted sequences for each gene are listed as in Table 1. *scr*, scrambled. *B*, control and knockdown cells were transduced with NF-κB luciferase lentivector to develop stable NF-κB reporter cell lines. TNF-α-, cFLIPs-, vFLIP-, and Tax-induced NF-KB activation levels were then evaluated as described in Fig. 3, *B* and *D*. Values shown are the mean ± S.D. (*error bars*) of the relative luciferase activity from five independent experiments. *C*, a stretch of hydrophobic amino acid (*AA*) residues on death effector domain 2 of cFLIP that is critical for interaction with FADD is highly conserved in the KSHV (*KS*) vFLIP. Sequence alignment was performed by PRALINE software. *D*, mutating the critical amino acids of this hydrophobic patch (Phe-114, Leu-115 in cFLIPs and Phe-115, Leu-116 in vFLIP) to alanine residues abrogated NF-κB activation by cFLIP variants but not vFLIP. NF-κB luciferase reporter assays were carried out in HEK293T cells as described in Fig. 5*C. E*, co-immunoprecipitation assays showing that F114A/L115A mutants of cFLIP variants are unable to associate with the FADD·RIP1 complex. HEK293T cells (seeded in 6-well plates) were transfected with 1 μg of HA-FADD vector together with 1 μg of pcDNA3 constructs expressing cFLIP variants or vFLIP. 48 h later, cell lysates were extracted and immunoprecipitated (*IP*) with 0.5 μg of anti-HA or an isotype-matched control antibody (*C*). Cell lysates and the immunoprecipitates were then analyzed by Western blotting with the indicated antibodies. * indicates the position of p43-FLIP bands.

To further probe the mechanism of action of cFLIP_S_ and p22-FLIP, we generated HEK293T cells that were knocked down for other molecules reported to be present in complexes with cFLIP isoforms or vFLIP (Fig. 6*A*). Atg3 has been reported to interact directly with vFLIP and cFLIPs (34), FADD interacts with cFLIP isoforms (for a review, see Ref. 2), and RIP1 interacts with FADD (35). Fig. 6*B* shows that FADD and RIP1 were both required for cFLIP_S_ and p22-FLIP activation of IKK, whereas Atg3 and caspase-8 were dispensable. A hydrophobic stretch of amino acids in cFLIP has been identified as critical for its interaction with FADD (4), and a similar sequence can be found in vFLIP (Fig. 6*C*). We mutated critical amino acids in this hydrophobic stretch, which resulted in blocking IKK activation by cFLIP_S_, p22-FLIP, and cFLIP_L_ but not vFLIP (Fig. 6*D*). We then demonstrated that this mutation prevented interaction of the cFLIP isoforms with FADD, whereas FADD and RIP1 were present in these cells as a preassembled complex (Fig. 6*E*). These data provide an explanation for the lack of inhibition of cFLIP_S_ and p22-FLIP by CYLD as ubiquitinated RIP1 bound to IKKγ has been reported to be protected from deubiquitination (36). Although mutation of the putative FADD binding domain in cFLIP_L_ prevented activation of IKK (Fig. 6*D*), cFLIP_L_ did not require FADD or RIP1 for its action (Fig. 6*B*), suggesting that this region interacts with a different effector to trigger LUBAC activity. This effector may be TRAF2, which has been reported to interact with the N-terminal region of cFLIP_L_ and to be required for NF-κB activation (6). Our finding that RIP1 was not required for cFLIP_L_ activation differs from the report of its role in T cell receptor-triggered NF-κB activation where RIP1 was required (11).

**FIGURE 7.**
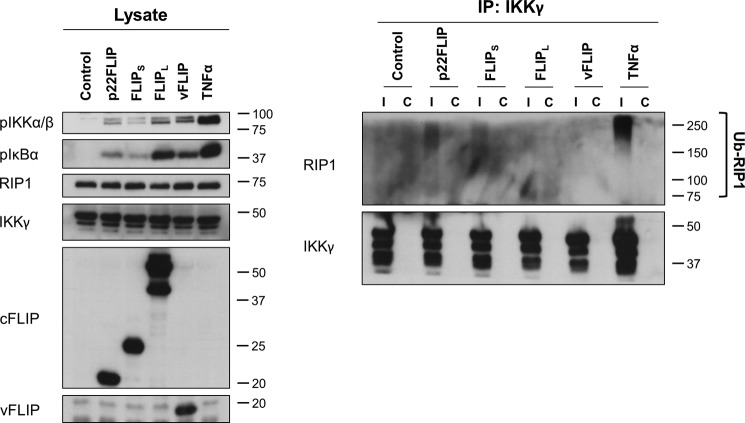
**Overexpression of p22-FLIP and cFLIP_S_ results in RIP1 ubiquitination and its association with IKKγ.** HEK293 cells were transduced with cFLIP variant or vFLIP lentivector. After 48 h, cells were lysed in a buffer containing 10 mm *N*-ethylmaleimide and 20 mm iodoacetamide to inhibit deubiquitinases. As a positive control, cells were treated with TNF-α (250 ng/ml) for 5 min. The cell lysates were immunoprecipitated (*IP*) with 2 μg of an anti-IKKγ (Ι) or an isotype-matched control antibody (*C*). The immunoprecipitates were analyzed by immunoblotting using the antibodies shown. Results are representative of two independent experiments. *Ub*, ubiquitin.

To further examine the role of RIP1 in IKK activation by cFLIP_S_ and p22-FLIP, we then examined ubiquitinated RIP1 binding to IKKγ. Fig. 7 shows that both cFLIP_S_ and p22-FLIP induced ubiquitinated RIP1 binding to IKKγ, whereas cFLIP_L_ and vFLIP did not.

##### Recombinant vFLIP Can Directly Activate IKK When Added to Cell Lysate

**FIGURE 8.**
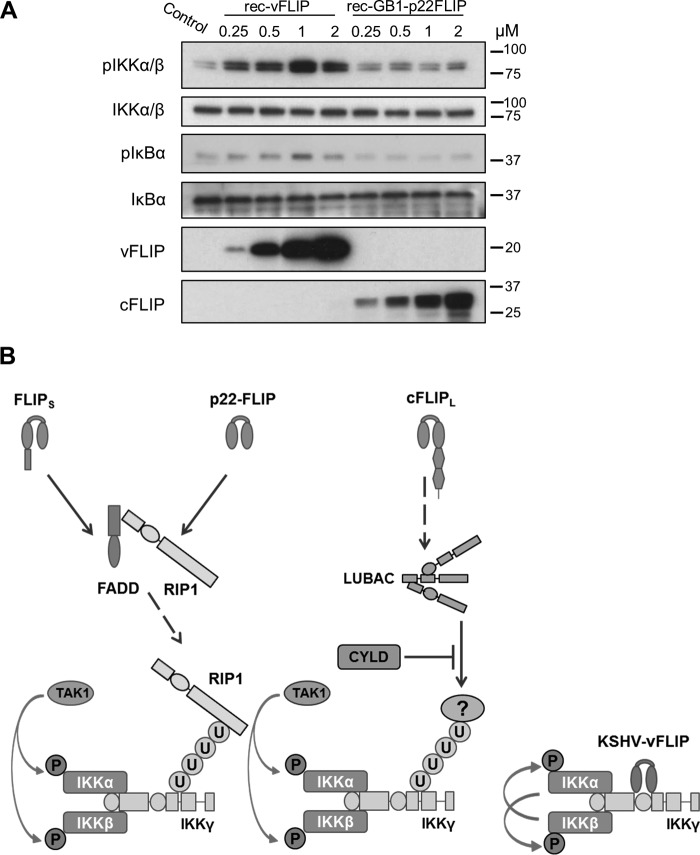
**Recombinant KSHV vFLIP activates IKK when added to cell lysate.** *A*, IKK activation by recombinant (*rec*) vFLIP or recombinant p22-FLIP was analyzed using an *in vitro* IκBα phosphorylation assay. Briefly, HEK293T cells were lysed in HTX lysis buffer, and the S100 fractions were extracted. The lysates were then incubated with an ATP-generating buffer and increasing concentrations of recombinant vFLIP or p22-FLIP at 37 °C for 10 min. Activation of the IKK complex was detected by immunoblotting to detect phospho-IκBα and phospho-IKKα/β. The blot was reprobed for total IKKα/β to ensure equal loading of protein. *B*, a model of IKK activation by cFLIP isoforms and KSHV vFLIP. *U*, ubiquitin.

As vFLIP and cFLIP isoforms had very different requirements for ubiquitination and TAK1 in their activation of IKK, we then produced recombinant vFLIP and p22-FLIP proteins and added them to HEK293T cell lysate. Fig. 8*A* shows that recombinant vFLIP induced phosphorylation of the kinase subunits of IKK and that IKK became activated in the lysate as IκBα was phosphorylated. This occurred within 10 min of incubation with the lysate; in contrast, recombinant p22-FLIP could not activate IKK under these conditions (Fig. 8*A*). Similar direct activation of the IKK kinase in cell lysates supplemented with recombinant Tax has been reported (37).

The results from this study allowed us to formulate a model of how vFLIP and cFLIP isoforms activate IKK (Fig. 8*B*). We propose that vFLIP binds directly to IKKγ and causes activation of the kinase subunits by stimulating autophosphorylation independently of upstream ubiquitination or kinases. In contrast, cFLIP_S_ and p22-FLIP are found in a complex with FADD and RIP1. As cFLIP_S_ and p22-FLIP could not be detected bound to IKK (Fig. 3), we suggest that ubiquitinated RIP1 is generated by and then released from this complex. Ubiquitinated RIP1 then binds directly to the ubiquitin binding domain of IKKγ, recruits TAK1, and activates IKK. However, cFLIP_L_ requires LUBAC to generate a different, as yet unidentified, ubiquitinated substrate, which then activates IKK.

**FIGURE 9.**
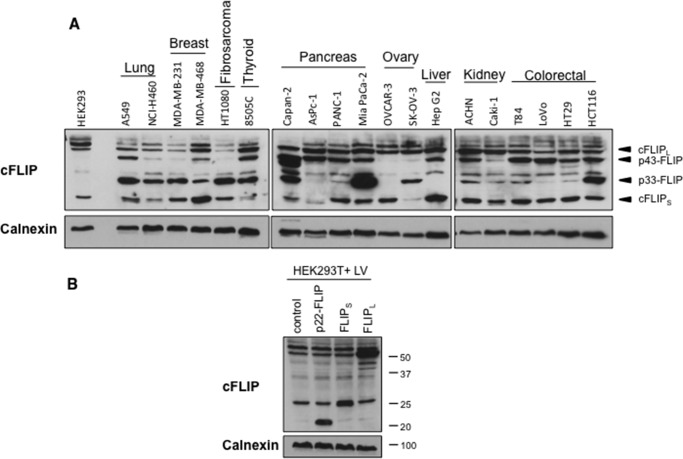
**cFLIP isoforms are overexpressed in human tumor cell lines.** *A*, immunoblotting analysis of expression levels of cFLIP variants in human tumor cells. Cytoplasmic extracts were prepared, normalized for protein content, then separated by SDS-PAGE, and immunoblotted using the anti-cFLIP antibody G11 and anti-calnexin as a loading control. *B*, comparing the endogenous and lentivector (*LV*)-expressed levels of cFLIP isoforms in HEK293T cells. Cells were transduced with lentivectors encoding GFP, p22-FLIP, cFLIP_S_, or cFLIP_L_, and immunoblotting analysis was performed as described in *A*.

In many of these experiments, we had constitutively expressed cFLIP isoforms using lentivectors. To test whether this mimicked physiological expression levels of cFLIP, we examined the level of endogenous cFLIP isoform expression in an extensive panel of human tumor cells. Fig. 9*A* shows that all the cells we tested expressed cFLIP_L_ and at least one shorter cFLIP variant; we did not detect p22-FLIP in any cell. For these experiments, we changed the cFLIP antibody to the monoclonal G11, which is considerably more sensitive than the monoclonal NF6 for the detection of short human cFLIP variants. Fig. 9*B* shows that the level of cFLIP_S_ and cFLIP_L_ achieved by lentivector transduction of HEK293T cells was a modest augmentation of the basal level expressed by these cells and similar to that seen in human tumor cells. These findings are compatible with the observation that 293T cells display a significant basal level of IKK activation (Figs. 3 and 8).

## Discussion

These data show that all cFLIP variants can activate IKK via a transient interaction of a ubiquitinated protein with the ubiquitin binding domain of IKKγ. The cFLIP_L_ isoform requires the LUBAC ubiquitination complex but does not require caspase-8 or cFLIP_L_ cleavage. In contrast, cFLIP_S_ and p22-FLIP form a stable complex with FADD and RIP1, which are required for IKK activation. cFLIP_L_ was previously reported to activate IKK following CD95 stimulation by the binding of a p43 fragment to IKK (6), and another study reported that the p22-FLIP cleavage product interacts directly with NEMO/IKKγ to activate IKK (7). We have demonstrated that IKK activation following cFLIP_L_ overexpression does not require cFLIP_L_ cleavage. Furthermore, our evidence for different pathways of NF-κB activation demonstrates that p22-FLIP is not simply the effector of cFLIP_L_, although cFLIP_L_ cleavage to generate p22-FLIP may switch the mechanism of NF-κB activation.

The cFLIP_S_·FADD·RIP1 or p22-FLIP·FADD·RIP1 complexes are reminiscent of a cytoplasmic complex, termed the ripoptosome, induced by treatments of human tumor cells that deplete or inhibit *cellular inhibitor of* apoptosis proteins (38, 39). This depletion leads to the formation of a RIP1·FADD·procaspase-8 complex. *Cellular inhibitors of* apoptosis inhibit formation of this complex by ubiquitinating RIP1. In the absence of FLIP proteins, procaspase-8 molecules homodimerize in the ripoptosome, promoting cell death by apoptosis. Recruitment of cFLIP_L_ to this complex leads to partial caspase-8 activation, resulting in cell survival (40). In contrast, cFLIP_S_ has been reported to block caspase-8 activation and facilitate cell death through necroptosis (39). A complex containing cFLIP_L_, RIP1, FADD, caspase-8, and TRADD (tumor necrosis factor receptor 1-associated death domain) is induced by TNF receptor signaling (41); a complex containing RIP1, FADD, caspase-8, and IKKγ has been detected after TRAIL triggering (42), and a cFLIP·RIP1·FADD·caspase-8 complex has been found after Fas signaling (43). Our data show that constitutive expression of cFLIP_S_ or p22-FLIP leads to formation of a FLIP·FADD·RIP1 complex that leads to NF-κB activation. The relationship between other complexes and this NF-κB-activating complex remains to be determined, but we have clearly shown that caspase-8 is not required for NF-κB activation by cFLIP_S_ or p22-FLIP.

The expression of cFLIP proteins is tightly regulated in normal cells. At the transcriptional level, cFLIP isoforms are up-regulated by NF-κB activation so they positively regulate their own expression (44). cFLIP_S_ is particularly more prone to ubiquitination and displays a considerably shorter half-life compared with cFLIP_L_ (5). This is largely due to the unique 19-amino acid C-terminal sequence of cFLIP_S_ that is ubiquitinated at lysine residues 192 and 195; the stability of cFLIP_R_ is similar to cFLIP_S_ (3). p22-FLIP is more stable than cFLIP_S_ as it lacks this C-terminal sequence (7). By using overexpression of cFLIP in our experiments, we have over-ridden some of these control mechanisms; however, there is evidence for overexpression of cFLIP isoforms in tumor cells (for a review, see Ref. 45), which is the situation that we have mimicked.

Up-regulation of cFLIP expression has been reported in many types of human solid tumors and lymphomas. We have confirmed these finding and shown that diverse human tumor cell types express a variety of cFLIP isoforms. There is also evidence that the level of cFLIP may determine clinical outcome (46–49). cFLIP inhibition or down-regulation has therefore been proposed as a potential cancer therapy with the assumption that this will operate by sensitizing tumor cells to Fas-, TRAIL-, or TNF-induced cell death (for a review, see Ref. 45). However, tumor cells may also be dependent on cFLIP activation of NF-KB for their survival (49). Our dissection of the different mechanisms of cFLIP activation of IKK will allow the development of reagents to inhibit these pathways selectively and thereby assess their role in cFLIP-driven tumorigenesis.

## Author Contributions

M. B. designed and performed experiments and co-wrote the paper. C. A. D. and A. S. designed and created reagents. D. E. designed experiments. C. B. designed and performed experiments. T. B. and M. K. C. designed experiments and co-wrote the paper.

## Footnotes

2 The abbreviations used are:

FLICE: FADD-like interleukin-1β-converting enzyme
FLIP: FLICE-like inhibitory protein
IKK: inhibitor of NF-κB kinase
cFLIP: cellular FLICE-like inhibitory protein
FADD: Fas-associated death domain
LUBAC: *linear ubiquitin chain assembly complex*
*vFLIP*: viral FLIP
cFLIP_L_: long isoform of cFLIP
cFLIP_S_: short isoform of cFLIP
cFLIP_R_: cFLIP Raji
DR: death receptor
KSHV: Kaposi sarcoma-associated herpesvirus
pIκBα: phospho-IκBα
pIKKα/β: phospho-IKKα/β
TRAIL: TNF-related apoptosis-inducing ligand
NEMO: NFκB essential modifier
HOIP: heme-oxidized iron-responsive element-binding protein 2 ubiquitin ligase-1
CYLD: cylindromatosis
SHARPIN: shank-associated RH domain-interacting protein.
